# Dexamethasone Sensitizes Cancer Stem Cells to Gemcitabine and 5-Fluorouracil by Increasing Reactive Oxygen Species Production through NRF2 Reduction

**DOI:** 10.3390/life11090885

**Published:** 2021-08-27

**Authors:** Shuhei Suzuki, Masahiro Yamamoto, Tomomi Sanomachi, Keita Togashi, Asuka Sugai, Shizuka Seino, Takashi Yoshioka, Masashi Okada, Chifumi Kitanaka

**Affiliations:** 1 Department of Molecular Cancer Science, Yamagata University School of Medicine, Yamagata 990-9585, Japan; s-suzuki@med.id.yamagata-u.ac.jp (S.S.); sanomachi2000t@gmail.com (T.S.); ke-togashi@med.id.yamagata-u.ac.jp (K.T.); s-asuka@med.id.yamagata-u.ac.jp (A.S.); s.sizuka@med.id.yamagata-u.ac.jp (S.S.); m-okada@med.id.yamagata-u.ac.jp (M.O.); 2 Department of Clinical Oncology, Yamagata University School of Medicine, Yamagata 990-9585, Japan; ytakashi@med.id.yamagata-u.ac.jp; 3 Department of Ophthalmology and Visual Sciences, Yamagata University School of Medicine, Yamagata 990-9585, Japan; 4 Research Institute for Promotion of Medical Sciences, Yamagata University Faculty of Medicine, Yamagata 990-9585, Japan

**Keywords:** ROS, cancer stem cell, dexamethasone

## Abstract

Cancer stem cells (CSCs) have high tumor-initiating capacity and are resistant to chemotherapeutic reagents; thus eliminating CSCs is essential to improving the prognosis. Recently, we reported that dexamethasone increases the effects of gemcitabine on pancreatic CSCs; however, the mechanism involved remains to be fully elucidated. In this study, we explored the role of reactive oxygen species (ROS) in the dexamethasone-induced chemosensitization of CSCs. Dexamethasone increased the growth-inhibitory effects of gemcitabine and 5-fluorouracil, whereas N-acetyl-cysteine, a ROS scavenger, abolished this effect. Although dexamethasone alone did not increase ROS levels, dexamethasone promoted the increase in ROS levels induced by gemcitabine and 5-fluorouracil. Dexamethasone treatment reduced the expression of NRF2, a key regulator of antioxidant responses, which was attenuated by siRNA-mediated knockdown of the glucocorticoid receptor. Furthermore, brusatol, a suppressor of NRF2, sensitized pancreatic CSCs to gemcitabine and 5-fluorouracil. Of note, essentially, the same mechanism was functional in ovarian and colon CSCs treated by the combination of dexamethasone and chemotherapeutic agents. Our study suggests that dexamethasone can sensitize CSCs to chemotherapeutic agents by promoting chemotherapy-induced ROS production through suppressing NRF2 expression.

## 1. Introduction

Cancer is one of the most common causes of death [1]. Although a number of clinical trials have been conducted to date, the prognosis of cancer remains poor. Thus, there is an urgent need to develop novel treatment strategies to improve the prognosis of cancer patients.

Cancer stem cells (CSCs) are a subpopulation of tumor cells that have high tumor initiation capacity and chemoresistance. CSCs play important roles in recurrence and metastasis. Furthermore, recent advances in research on circulating tumor cells (CTCs) by liquid biopsy demonstrated that CTCs are a heterogeneous population of cells containing CTCs showing CSC-like properties, termed circulating CSCs (CCSCs), which contribute to distant metastasis [2,3]. Therefore, CSCs are considered one of the most promising targets to achieve a cure. However, it is challenging to eliminate CSCs due to their chemoresistance [4,5,6,7]. Mechanisms underlying the chemoresistance of CSCs include the ability to eliminate drugs, slow cell proliferation rate, highly efficient DNA repair, epithelial-mesenchymal transition (EMT), resistance to apoptosis, and suppression of cellular reactive oxygen species (ROS) [8,9,10]. Although these mechanisms are being targeted in drug development, none of the drugs have been clinically approved.

Drug repositioning/repurposing is a strategy for identifying new uses for already-approved drugs [11]. As the safety profile and therapeutically effective doses of already-approved drugs are well-characterized, this strategy is effective in reducing the time and cost associated with the processes of drug development. Dexamethasone is a glucocorticoid used as an antiemetic in cancer patients and has a well-established safety profile [12,13]. Notably, previous studies demonstrated that glucocorticoids sensitize non-cancer stem cells of several types of cancers, such as colorectal, breast, lung, liver, and pancreatic cancer, to chemotherapy [14,15,16,17]. We also reported that dexamethasone targets pancreatic CSCs and sensitizes pancreatic CSCs to chemotherapeutic agents, such as gemcitabine, by suppressing the expression of survivin [18]. However, other than this particular mechanism, there are no other known mechanisms that explain how dexamethasone sensitizes CSCs to chemotherapeutic agents.

In the present study, we revealed that dexamethasone sensitizes CSCs to gemcitabine and 5-fluorouracil (5-FU) through a mechanism involving ROS.

## 2. Materials and Methods

### 2.1. Antibodies and Drugs

Anti-β-actin antibody (A1978) was purchased from Sigma-Aldrich (St. Louis, MO, USA), and anti-nuclear factor erythroid 2-related factor 2 (NRF2) antibody (#12721), anti-glucocorticoid receptor (GR) antibody (#12401), anti-phospho-c-Jun antibody (#9261), anti-c-Jun antibody (#9165), and anti-survivin antibody (#2808) were purchased from Cell Signaling Technology, Inc. (Beverly, MA, USA). Dexamethasone (Fuji Pharma Co., Ltd., Tokyo, Japan) was dissolved in phosphate-buffered saline (PBS) to prepare a 1 mM stock solution. 5-FU, gemcitabine, 2′,7′-dichlorofluorescein diacetate (DCF-DA), N-acetyl-cysteine (NAC), and brusatol were purchased from Sigma-Aldrich, and SP600125 was purchased from Merck Millipore (Darmstadt, Germany). They were dissolved in dimethyl sulfoxide (DMSO) to prepare 200 mM 5-FU, 1 mM gemcitabine, 20 mM DCF-DA, 5 M NAC, and 50 mM brusatol stock solutions.

### 2.2. Cell Culture

The cancer stem-like cell (CSLC) lines used in this study (PANC-1 CSLC, PSN-1 CSLC, WiDr CSLC, and A2780 CSLC) were established from PANC-1, PSN-1, WiDr, and A2780, respectively, using previously published protocols [19,20,21]. Briefly, cells were cultured on non-coated dishes in stem cell culture media described below. Cells from spheres formed under this culture condition were transferred and amplified under the monolayer stem cell culture condition. To enrich cancer stem-like cells, the cells were implanted subcutaneously into nude mice before (for PANC-1 CSLC and PSN-1 CSLC) or after (for A2780 CSLC) the sphere formation, and cells dissociated from the tumors were cultured in non-coated dishes in stem cell culture medium. The established CSLC lines have been characterized by the expression of stem cell markers, such as CD133 and Sox2, as well as a sphere-forming ability [19,20,21]. The pancreatic cancer cell line PANC-1 was made available by the Cell Resource Center for Biomedical Research, Institute of Development, Aging and Cancer, Tohoku University, and PSN-1 was kindly donated by Dr. T. Yoshida at the National Cancer Center Research Institute, who established the cell line [22]. The ovarian cancer cell line A2780 was kindly donated by Dr. T. Tsuruo at the Institute of Molecular and Cellular Biosciences, the University of Tokyo, and Drs. RF Ozols and TC Hamilton at the National Institute of Health [23,24]. The colorectal cancer cell line WiDr was purchased from the Tsukuba Resource Center (Tsukuba, Japan). For each CSLC line, the short tandem repeat (STR) was genotyped (Bio-synthesis, Lewisville, TX, USA), and the information was compared with the American Type Culture Collection (ATCC) STR database to ensure that the correct genomic sequence was obtained. CSLC cells were cultured in a monolayer under conventional culture conditions for CSCs [19]. Specifically, cells were cultured in a collagen I-coated dish (Iwaki, Tokyo, Japan) with stem cell culture media consisting of DMEM/F12, 1% B27 (Gibco-BRL, Carlsbad, CA, USA), 26.2 mM D-(+)-glucose, 4.5 mM L-glutamine, 100 U/mL of penicillin, and 100 μg/mL of streptomycin. The cell media was changed every 3 days, and 20 ng/mL of epidermal growth factor (EGF) and fibroblast growth factor 2 (FGF2) (Peprotech, Rocky Hill, NJ, USA) were added to the culture media every day.

### 2.3. siRNA

Human GR alpha (NR3C1; #2 HSS178979, #3 HSS178980) siRNAs were used to knockdown GR, and Stealth RNAi^TM^ siRNA Negative Control Duplexes, Medium GC Duplex #2 (siCT) (Thermo Fisher Scientific, Waltham, MA, USA) was used as a negative control. RNAi was induced using Lipofectamine RNAiMAX^TM^ (Invitrogen Life Technologies, Carlsbad, CA, USA) as per the vendor’s protocol.

### 2.4. Cell Number and Viability

Cells that were pretreated with siRNA were subsequently washed and counted. The number of cells was kept consistent in all experiments. The number of live and dead cells was measured using the trypan blue exclusion test. Specifically, 0.4% trypan blue was mixed with an equal volume of cell suspension, and cell viability (%) was calculated by the following formula: 100 × (number of live cells/(number of live cells + dead cells)). Cell viability was also examined by adding 1 mg/mL of propidium iodide (PI) and 10 mg/mL of Hoechst 33342 to the culture medium and incubating for 10 min at 37 °C. The numbers of PI-positive cells and Hoechst 33342-positive cells were counted on fluorescence microscopy, and cell death was expressed as the ratio of PI-positive cells (dead cells) to Hoechst 33342-positive cells (all cells). 

### 2.5. Quantification of Intracellular ROS 

Cells were incubated with 10 μM DCF-DA in the dark for 30 min at 37 °C and washed twice with PBS. Stained cells (1 × 10^4^ cells/ sample) were subsequently analyzed by flow cytometry (FACSCanto^TM^ II Flow Cytometer, BD Biosciences, Franklin Lakes, NJ, USA). Cell debris and cell aggregates were eliminated by forward and side scatters. Cells exhibiting a signal above the threshold determined by unstained cells were considered DCF-DA-positive cells. The data were analyzed using Flow Jo version 7.6.5 (Treestar, Ashland, OR, USA).

### 2.6. Western Blot

The cells were washed in ice-cold PBS and dissolved in radioimmunoprecipitation assay (RIPA) buffer, which consisted of 10 mM Tris-HCl (pH 7.4), 0.1% SDS, 0.1% sodium deoxycholate, 1% NP-40, 150 mM sodium chloride, 1 mM ethylenediaminetetraacetic acid (EDTA), 1.5 mM sodium orthovanadate (V), 10 mM sodium fluoride, 10 mM tetrasodium pyrophosphate, 10 mM disodium β-glycerophosphate pentahydrate, and 1% protease inhibitor cocktail set III (Calbiochem). The cell suspension was subsequently centrifuged for 10 min at 11,000 × *g* at 4 °C, and the supernatant was collected to measure the protein concentration using a BCA protein assay kit (Pierce Biotechnology, Rockford, IL, USA). An equal amount of protein was then loaded for sodium dodecyl sulfate-polyacrylamide gel electrophoresis (SDS-PAGE) and transferred to a polyvinylidene difluoride membrane. The membrane was treated with a primary antibody and a horseradish peroxidase (HRP)-labeled secondary antibody according to the vendors’ protocol. The antigen-antibody reaction was detected using Immobilon Western Chemiluminescent HRP Substrate (Millipore, Billerica, MA, USA), and densitometry was performed by analyzing the images on ImageJ 1.52a software (National Institutes of Health, Bethesda, MD, USA).

### 2.7. Quantification of Glutathione

An oxidized/reduced glutathione (GSSG/GSH) Quantification Kit (Dojindo, Kumamoto, Japan) was used according to the vendor’s protocol to quantify the amount of GSH [25]. Absorbance was measured using a microplate reader (Model 680, BioRad, Hercules, CA, USA). 

### 2.8. Statistical Analysis 

The Student’s *t*-test was used for all analyses. *p* < 0.05 (indicated with *) was considered significant. For multiple comparisons, the significance levels were adjusted by the Bonferroni method.

## 3. Results

### 3.1. Sensitization of CSLCs to Gemcitabine and 5-FU Is Induced by Dexamethasone and Reversed by N-Acetyl-Cysteine

**Figure 1:**
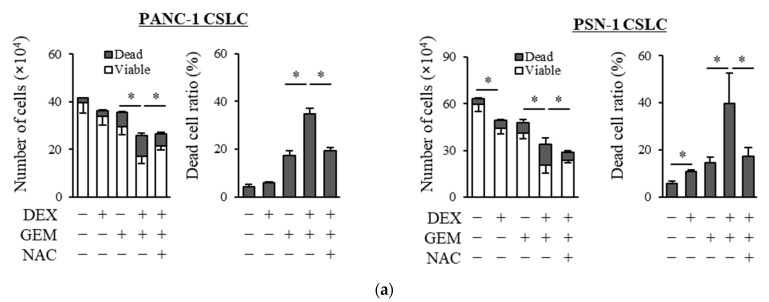

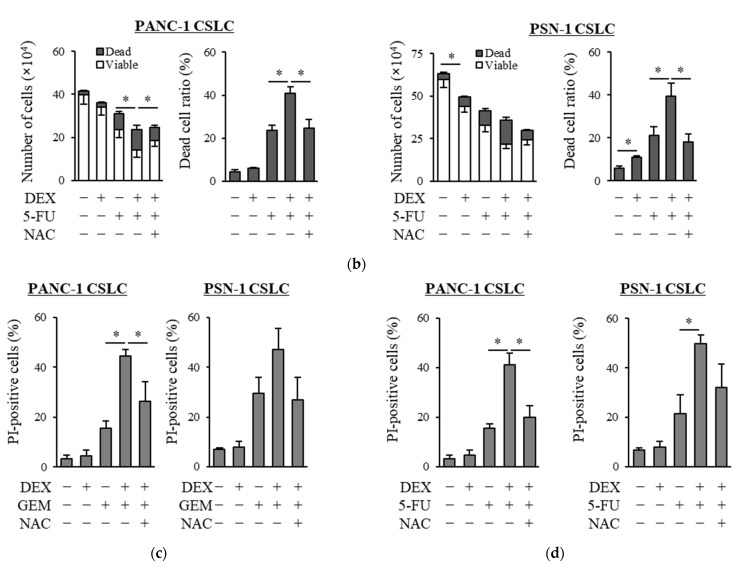
Dexamethasone sensitizes CSLCs to chemotherapy, and this effect is reversed by N-acetyl-cysteine: PANC-1 CSLC cells and PSN-1 CSLC cells were treated with dexamethasone for 6 days. Cells were then treated with 5 mM NAC for 10 min and subsequently treated with either gemcitabine (**a**,**c**) or 5-FU (**b**,**d**) for 3 days. After the treatments, cells were (**a**,**b**) stained with trypan blue and counted or (**c**,**d**) stained with PI and Hoechst 33342 and observed by fluorescence microscopy. Concentrations for each agent were as follows: 1 µM dexamethasone, 1 µM and 0.2 µM gemcitabine for PANC-1 CSLC and PSN-1 CSLC, respectively, 10 µM and 1 µM 5-FU for PANC-1 CSLC and PSN-1 CSLC, respectively, and 5 mM NAC. Values represent means + or − SD from triplicate samples of a representative experiment repeated twice with similar results. * *p* < 0.05.

We examined whether dexamethasone sensitizes pancreatic CSLCs (PANC-1 CSLC and PSN-1 CSLC), which are less sensitive to chemotherapeutic agents than parental non-CSCs [26], to gemcitabine and 5-FU. While dexamethasone pretreatment alone reduced the number of viable cells to a small extent, dexamethasone pretreatment of PANC-1 CSLC and PSN-1 CSLC cells prior to treatment with GEM or 5-FU substantially reduced the number of viable cells and increased the number of dead cells (Figure 1a,b). The increase in dead cells was also demonstrated by PI staining (Figure 1c,d). As gemcitabine and 5-FU exert their anti-cancer effects by inducing oxidative stress [24,25], we examined whether the effects of dexamethasone are altered by the addition of the antioxidant N-acetyl-cysteine (NAC). As shown in Figure 1a–d, the addition of NAC reduced the effects of dexamethasone on sensitizing pancreatic CSLCs to gemcitabine and 5-FU. Therefore, dexamethasone sensitizes pancreatic CSLCs to gemcitabine and 5-FU, and this effect may be induced by oxidative stress.

### 3.2. Dexamethasone Promotes the Gemcitabine- and 5-FU-Induced Increase in ROS Levels in CSLCs 

**Figure 2:**
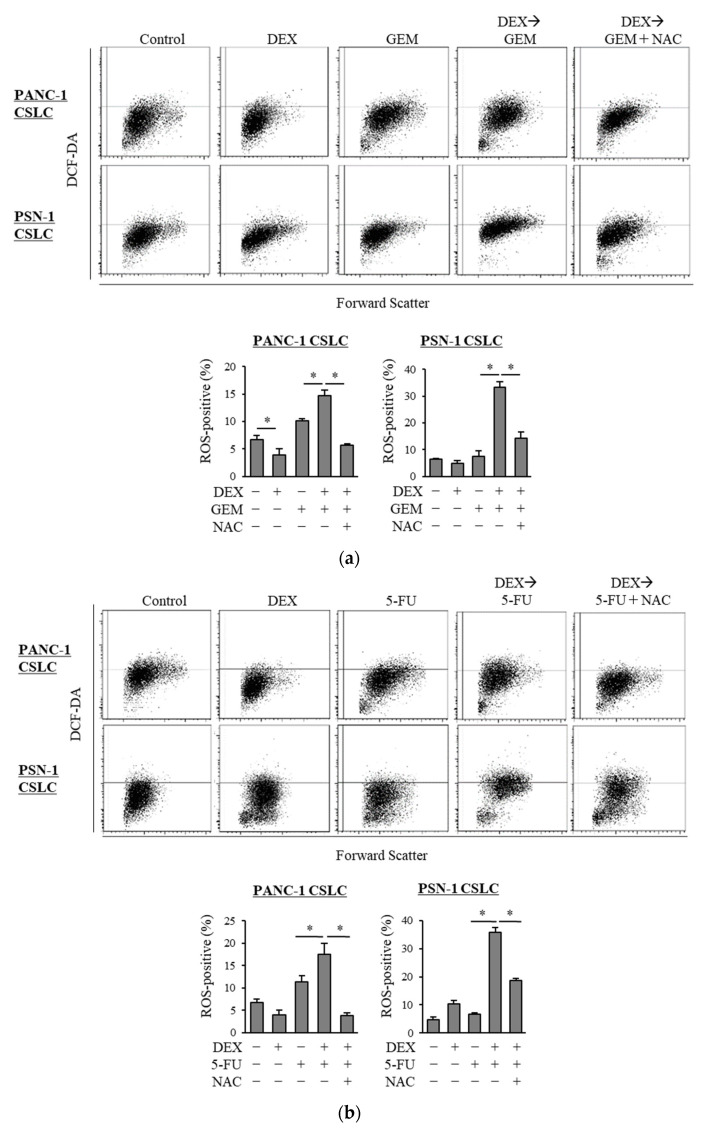
Dexamethasone increases the level of ROS in CSLCs after treatment with gemcitabine and 5-FU, and this effect is reversed by N-acetyl-cysteine: PANC-1 CSLC cells and PSN-1 CSLC cells were treated with dexamethasone for 6 days. Cells were then treated with 5 mM NAC for 10 min and subsequently treated with either gemcitabine (**a**) or 5-FU (**b**) for 3 days. Cells were then stained with DCF-DA and incubated for 30 min at 37 °C to measure the level of intracellular ROS using flow cytometry. Upper images show the scatter plots, and lower graphs show the percentage of ROS-positive cells for each treatment group. Concentrations of each agent were as follows: 1 µM dexamethasone, 1 µM and 0.2 µM gemcitabine for PANC-1 CSLC and PSN-1 CSLC, respectively, 10 µM and 1 µM 5-FU for PANC-1 CSLC and PSN-1 CSLC, respectively, and 5 mM NAC. Values represent means + SD from triplicate samples of a representative experiment repeated twice with similar results. * *p* < 0.05.

Based on our finding of the possible role of oxidative stress in the chemo-sensitizing effects of dexamethasone, we next examined the level of ROS in CSLCs when gemcitabine and 5-FU were combined with dexamethasone. In contrast to gemcitabine and 5-FU, which each increased the level of ROS in CSLCs, dexamethasone alone did not consistently increase the level of ROS. However, the pretreatment with dexamethasone prior to gemcitabine or 5-FU significantly increased the level of ROS (Figure 2a,b). Moreover, NAC inhibited the increase in the ROS levels induced by the addition of dexamethasone to gemcitabine and 5-FU (Figure 2a,b). Changes in the level of ROS that were induced by NAC, dexamethasone, gemcitabine, and 5-FU correlated with the anti-CSC effects of the respective combinations of the drugs, suggesting that the chemo-sensitizing effects of dexamethasone are induced by the increased ROS levels.

### 3.3. Dexamethasone Suppresses NRF2 Expression in CSLCs

**Figure 3:**
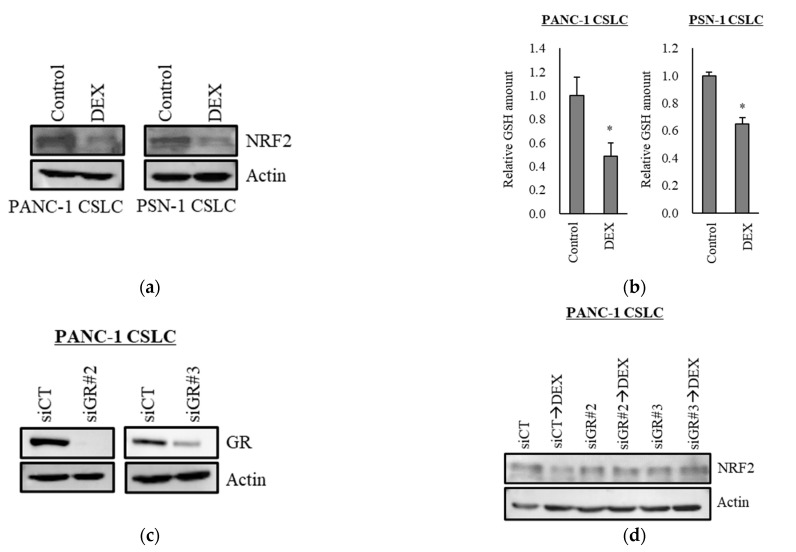

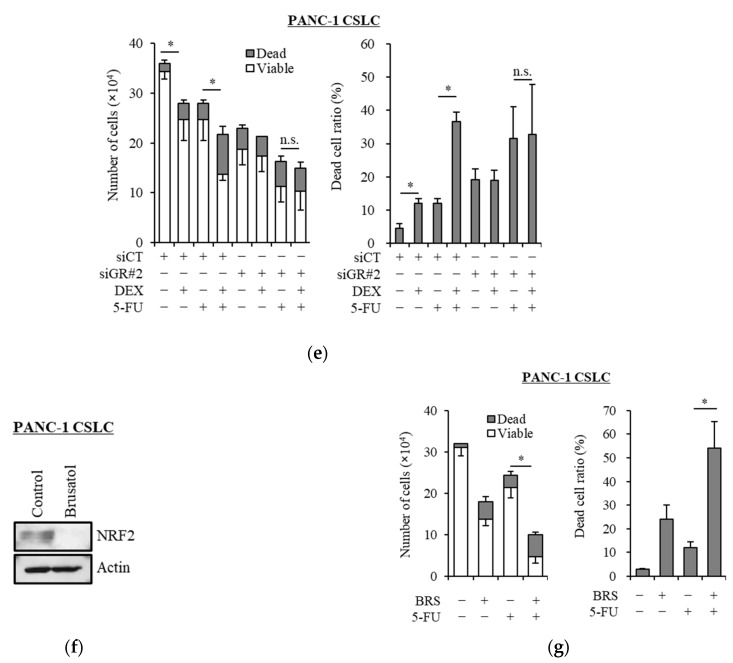
Dexamethasone sensitizes cells to chemotherapy by suppressing NRF2 expression via GR: (**a**) PANC-1 CSLC cells and PSN-1 CSLC cells were treated with dexamethasone for 6 days, and immunoblotting was subsequently performed to evaluate the level of protein. (**b**) PANC-1 CSLC cells and PSN-1 CSLC cells were treated with dexamethasone for 6 days, and GSH was quantified using a GSSG/GSH Quantification Kit, as described in the Materials and Methods. An untreated group was used as the control. (**c**,**d**) GR in PANC-1 CSLC cells was knocked-down with siRNA for 3 days, and cells were subsequently treated with 1 µM dexamethasone for 6 days. Immunoblotting was performed to evaluate the level of protein. (**e**) Cells pretreated with a siRNA against GR for 3 days were treated with dexamethasone for 6 days, followed by treatment with 5-FU for 3 days, and the number of cells was counted based on trypan blue staining. (**f**) Similarly, PANC-1 CSLC cells were treated with 0.25 µM brusatol for 3 days, and immunoblotting was performed to evaluate the level of protein. (**g**) Cells were treated with 0.25 µM brusatol (BRS) for 3 days, followed by treatment with 10 µM 5-FU for 3 days, and the number of cells was counted based on trypan blue staining. Values represent means + or − SD from triplicate samples of a representative experiment repeated twice with similar results. * *p* < 0.05, n.s.: not significant.

Transcription factor NRF2 is one of the known regulators of cellular responses to oxidative stress that controls the amount of intracellular ROS [27]. We, therefore, examined how the addition of dexamethasone impacts the expression of NRF2. As shown in Figure 3a, dexamethasone suppressed the expression of NRF2 in PANC-1 CSLC cells and PSN-1 CSLC cells. Furthermore, there was a significant decrease in GSH, a predominant intracellular antioxidant (Figure 3b). We then treated cells with dexamethasone after knocking down GR in PANC-1 CSLC cells to determine whether dexamethasone acts on GR to suppress NRF2. Knockdown of GR expression (Figure 3c) weakened the effects of dexamethasone on NRF2 suppression (Figure 3d) and resulted in the loss of dexamethasone-induced chemosensitivity (Figure 3e). Furthermore, suppression of NRF2 by the NRF2-inhibitor brusatol (Figure 3f) reduced the chemoresistance of PANC-1 CSLC cells (Figure 3g). This suggested that dexamethasone promotes chemotherapy-induced ROS production by suppressing the expression of NRF2, most likely via GR.

### 3.4. Effects of Dexamethasone on Colorectal Cancer and Ovarian CSLCs

**Figure 4:**
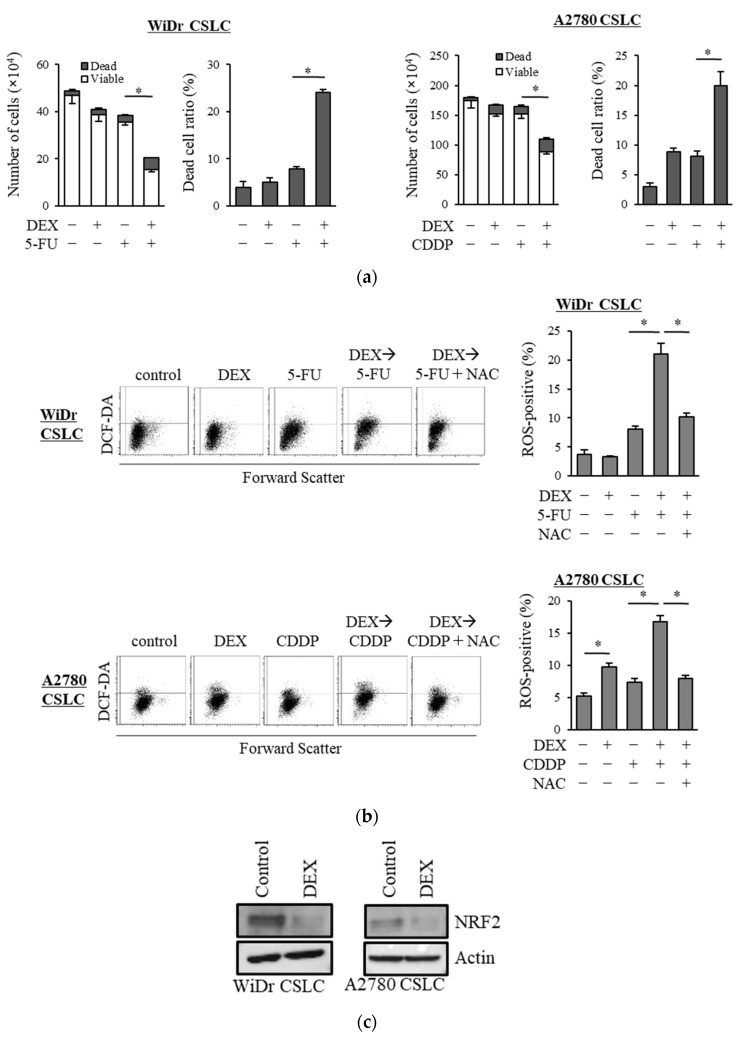
Results in the pancreatic CSLCs were reproduced in colorectal and ovarian CSLCs: (**a**) WiDr CSLC cells and A2780 CSLC cells were treated with dexamethasone for 6 days and subsequently treated with either 5-FU or cisplatin (CDDP) for 3 days. The number of cells was counted based on trypan blue staining to determine the viable cell number (left) and cell death (right). (**b**) WiDr CSLC cells (upper panel) and A2780 CSLC cells (lower panel) were treated with dexamethasone for 6 days. Cells were then treated with 5 mM NAC for 10 min and subsequently treated with either 5-FU or CDDP for 3 days. Cells were then stained with 10 µM DCF-DA and incubated for 30 min at 37 °C to measure the level of intracellular ROS using flow cytometry. Images on the left show the scatter plots, and graphs on the right show the percentage of ROS-positive cells for each treatment group. (**c**) WiDr CSLC cells and A2780 CSLC cells were treated with dexamethasone for 6 days. Immunoblotting was performed to evaluate the level of protein. Concentrations for each agent were as follows: 1 µM dexamethasone, 0.5 µM 5-FU, 20 µM CDDP, and 5 mM NAC. Values represent means + or − SD from triplicate samples of a representative experiment repeated twice with similar results. * *p* < 0.05.

We examined the chemo-sensitizing effects of dexamethasone on other CSLC lines and demonstrated that dexamethasone sensitized CSLCs established from the colon cancer cell line WiDr and ovarian cancer cell line A2780 to 5-FU and cisplatin, respectively (Figure 4a). Dexamethasone further increased the level of ROS induced by 5-FU and cisplatin in WiDr CSLC cells and A2780 CSLC cells, and NAC significantly reduced the ROS levels induced by the combination of dexamethasone with these chemotherapeutic agents (Figure 4b). Moreover, dexamethasone suppressed the expression of NRF2 in WiDr CSLC cells and A2780 CSLC cells (Figure 4c). These results were consistent with our findings in pancreatic CSLC cells and suggest that dexamethasone also sensitizes other CSLCs to chemotherapy by suppressing NRF2, thereby increasing the intracellular ROS levels.

## 4. Discussion

CSCs are a subpopulation of tumor cells within a tumor and are characterized by high tumor initiation capacity and chemoresistance. As they play important roles in tumor recurrence and metastasis, they have a significant impact on the prognosis of patients [5,8]. However, the chemoresistance of CSCs makes it challenging to eliminate them [8]. In the present study, we demonstrated that dexamethasone sensitizes CSLCs to gemcitabine and 5-FU through the inhibition of NRF2 expression, which promoted the increase in ROS levels induced by these drugs.

ROS production is one of the mechanisms that regulate the cytotoxicity of chemotherapeutic agents, such as gemcitabine and 5-FU [28,29]. We previously demonstrated that inhibitors of c-Jun N-terminal kinase (JNK) increase cellular production of ROS and sensitize cells to gemcitabine and 5-FU [26]. In the present study, we also found that dexamethasone increases the level of ROS in CSLCs, and sensitizes them to gemcitabine and 5-FU. Furthermore, the removal of ROS by NAC resulted in the reduction of the chemo-sensitizing effects of dexamethasone for gemcitabine and 5-FU. This suggests that the chemo-sensitizing effects of dexamethasone are mediated by its ability to regulate intracellular ROS levels.

NRF2 is a master activating transcription factor that induces the expression of antioxidant genes by reacting to ROS and binding to genetic sequences known as antioxidant response elements (AREs) [27]. In this study, dexamethasone suppressed the expression of NRF2 in CSLCs and reduced GSH. The expression of NRF2 was reversed when the expression of GR was knocked-down by siRNA. Consistent with our findings, previous studies on osteocytes and osteoblasts revealed that glucocorticoids reduce the expression of NRF2 and increase ROS production to induce apoptosis, suggesting that glucocorticoid-induced apoptosis plays a role in osteoporosis and osteonecrosis [30,31]. A study further demonstrated that GR directly binds to NRF2 and suppresses its transcription activity in hepatocytes [32]. In addition, Zhou et al. reported that flumethasone, a glucocorticoid agonist, suppresses the expression of NRF2 in lung cancer cells and sensitizes them to cisplatin, doxorubicin, and 5-FU [14]. Although dexamethasone reduced the expression of NRF2 and GSH in CSLCs in this study, treatment with it alone did not consistently increase the level of ROS. This suggests that rather than directly inducing ROS production, dexamethasone suppresses the expression of NRF2, which would otherwise eliminate and control the level of intracellular ROS. This will further promote the increase in ROS induced by gemcitabine and 5-FU and sensitize cells to these agents, whereas the reduced expression levels of NRF2 after dexamethasone treatment may nevertheless be sufficient to eliminate limited amounts of ROS endogenously produced by unstimulated (i.e., not treated with gemcitabine or 5-FU) cells. In accordance with the limited increase in ROS, the reduction of cell viability by dexamethasone treatment alone was also small. We previously reported that dexamethasone suppresses the expression of survivin via the JNK pathway to reduce chemoresistance in CSCs [18]. In the present study, we also assessed the effects of the JNK inhibitor SP600125 but found no significant change in the level of NRF2 (Supplementary Figure S1). Thus, the signaling pathway that mediates the effects of dexamethasone on NRF2 may be independent of the JNK pathway. Furthermore, the suppression of NRF2 by brusatol reduced survivin expression (Supplementary Figure S1), suggesting that the suppression of survivin by dexamethasone is also mediated by NRF2 reduction. Additional studies are needed to examine this in more detail. We also demonstrated that dexamethasone has similar effects in colorectal and ovarian CSCs. Therefore, in addition to pancreatic tumors, dexamethasone may also be effective in sensitizing tumor cells of other origins to chemotherapeutic agents that induce ROS production. 

Drug repositioning/repurposing is a strategy for identifying new uses for already-approved drugs and is effective in reducing the time and cost associated with the processes of drug development. Dexamethasone is currently used in cancer patients as an antiemetic [12,13], to prevent hypersensitivity [33], and to treat cerebral edema [34] and malignant bowel obstruction [35]. As the safety profile of dexamethasone is well-established in cancer patients, it may also be repurposed and used effectively as a chemo-sensitizing agent. Furthermore, it is inexpensive and may be cost-effective compared with other anti-cancer agents that are becoming increasingly expensive in recent years [36].

The prolonged administration of glucocorticoids can cause a number of side effects, such as insomnia, indigestion/discomfort in the upper abdomen, agitation, increased appetite, weight gain, acne, osteoporosis, and osteonecrosis [37,38,39]. In order to prevent these complications, recent studies recommend the administration of dexamethasone as an antiemetic in a shorter period of time in a regimen known as the dexamethasone-sparing regimen [40,41]. Our study suggests the need to consider the chemo-sensitizing effects of dexamethasone in determining the duration of dexamethasone therapy.

## 5. Conclusions

In conclusion, dexamethasone may be effective in overcoming the chemoresistance of CSCs by suppressing NRF2 and increasing ROS production.

## Data Availability

The data presented in this study are available in the supplementary materials.

## Supplementary Materials

The following are available online at https://www.mdpi.com/article/10.3390/life11090885/s1, Figure S1: Inhibition of NRF2 and JNK pathways: PANC-1 CSLC cells were treated with either 0.25 µM brusatol or 20 µM SP600125 for 3 days. Immunoblotting was performed to evaluate the level of protein. Figure S2: Membranes and densitometry readings/intensity ratio.
