# Supplementary material for: Dexamethasone Sensitizes Cancer Stem Cells to Gemcitabine and 5-Fluorouracil by Increasing Reactive Oxygen Species Production through NRF2 Reduction
Source: Life (Basel). 2021 Aug 27;11(9):885. doi: 10.3390/life11090885 (PMC8470402; doi:10.3390/life11090885)
Supplement: Supplementary file 1 [file life-11-00885-s001.zip › life-1295908-supplementary.pdf]

Supplementary Figure 1

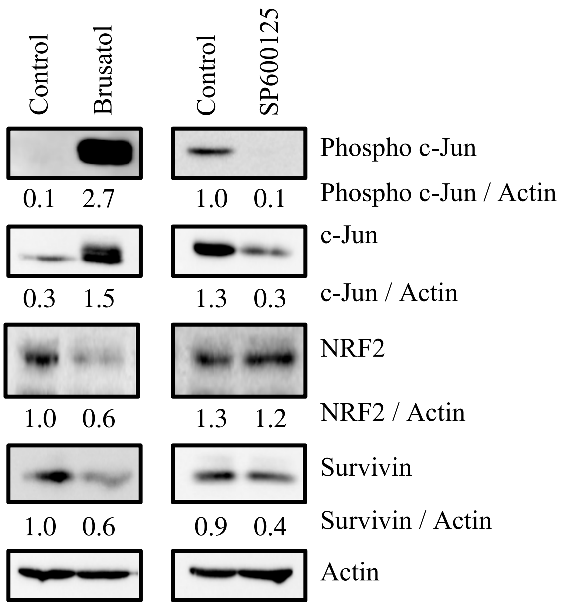

Supplementary Figure 2

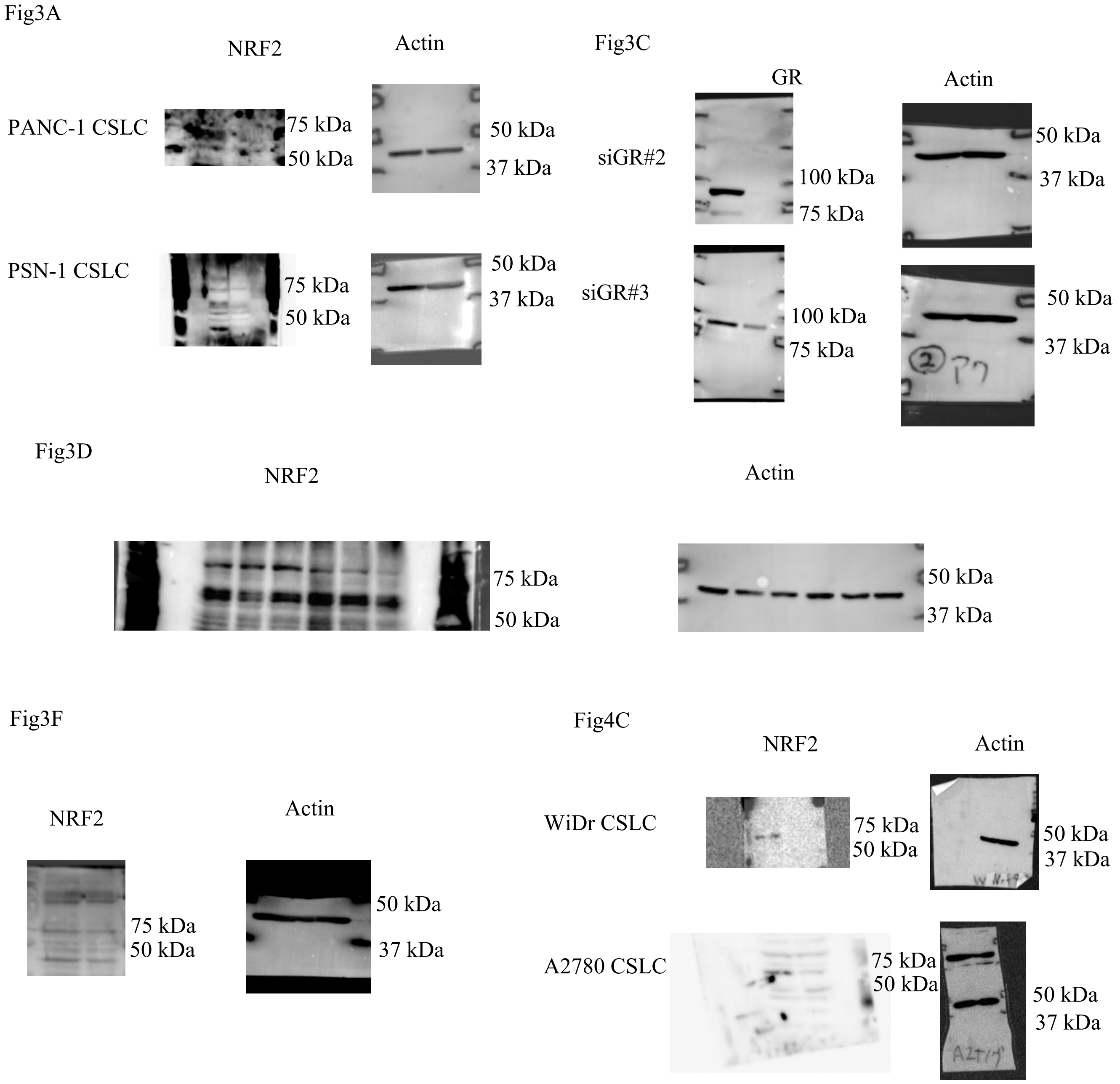

Supplementary Figure 2 (continued)

Supplementary Figure 1

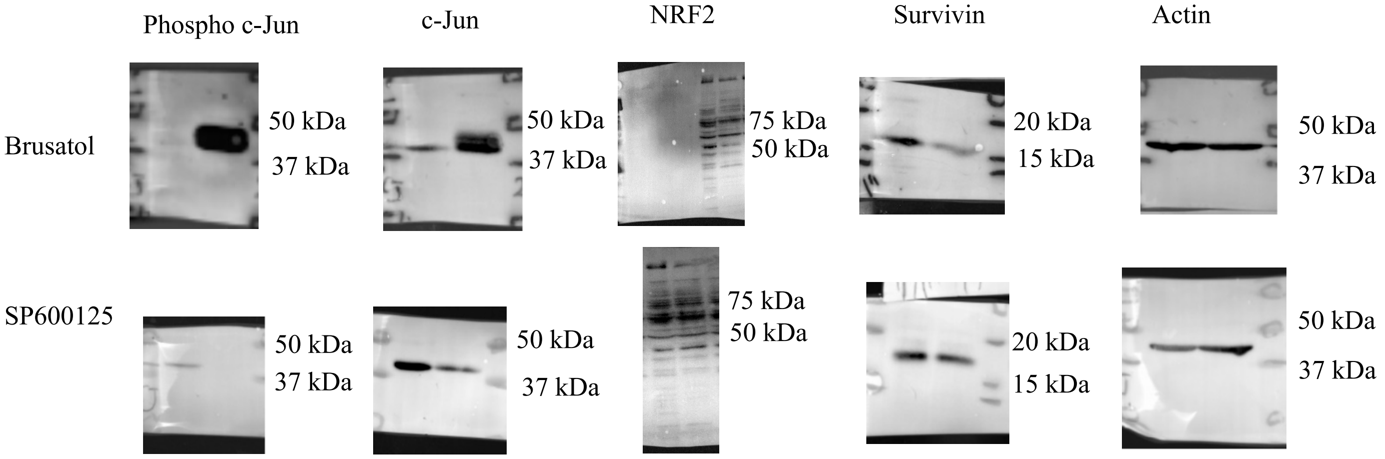

Densitometry readings/intensity ratio

Figure 3a				Figure 3c		
PANC-1 CSLC	Control	DEX			siCT	siGR#2
Actin	11634	10986		Actin	16329	15821
NRF2	20709	3735		GR	22480	1195
ratio	1.78	0.34		ratio	1.38	0.08
PSN-1 CSLC	Control	DEX			siCT	siGR#3
Actin	15846	16389		Actin	13905	13511
NRF2	13469	4917		GR	9117	3495
ratio	0.85	0.30		ratio	0.66	0.26
Figure 3d						
	siCT	siCT DEX	siGR#2	siGR#2 DEX	siGR#3	siGR#3 DEX
Actin	9637	12302	12932	12069	13417	14025
NRF2	5410	2891	8147	7362	7145	8405
ratio	0.56	0.24	0.63	0.61	0.53	0.60
Figure 3f				Figure 4c		
	Control	Brusatol		WiDr CSLC	Control	DEX
Actin	14632	15392		Actin	14442	15236
NRF2	5707	924		NRF2	17909	3961
ratio	0.39	0.06		ratio	1.24	0.26
				A2780 CSLC	Control	DEX
				Actin	13217	13698
				NRF2	8720	2192
				ratio	0.66	0.16
Sup Figure 1				Sup Figure 1		
	Control	Brusatol			Control	SP600125
Actin	16392	14832		Actin	10923	13659
P-c-Jun	1322	39749		P-c-Jun	6162	542
ratio	0.08	2.68		ratio	0.56	0.04
c-Jun	4269	32338		c-Jun	14419	3005
ratio	0.26	2.18		ratio	1.32	0.22
NRF2	7541	1632		NRF2	8519	7649
ratio	0.46	0.11		ratio	0.78	0.56
Survivin	10819	3856		Survivin	11644	8059
ratio	0.66	0.26		ratio	1.07	0.59
